# Reduced 30-day in-hospital but increased long-term mortality for weekend vs weekday acute medical admission

**DOI:** 10.1007/s11845-024-03729-y

**Published:** 2024-06-11

**Authors:** Richard Conway, Candice Low, Declan Byrne, Deirdre O’Riordan, Bernard Silke

**Affiliations:** 1 Trinity College Dublin, The University of Dublin Trinity College, Dublin, Ireland; 2 St. James’s Hospital, Dublin, Ireland

**Keywords:** 30-Day mortality, Long-term survival, Weekend

## Abstract

**Background:**

Acute medical admission at the weekend has been reported to be associated with increased mortality. We aimed to assess 30-day in-hospital mortality and subsequent follow-up of all community deaths following discharge for acute medical admission to our institution over 21 years.

**Methods:**

We employed a database of all acute medical admissions to our institution over 21 years (2002–2023). We compared 30-day in-hospital mortality by weekend (Saturday/Sunday) or weekday (Tuesday/Wednesday) admission. Outcome post-discharge was determined from the National Death Register to December 2021. Predictors of 30-day in-hospital and long-term mortality were analysed by logistic regression or Cox proportional hazards models.

**Results:**

The study population consisted of 109,232 admissions in 57,059 patients. A weekend admission was associated with a reduced 30-day in-hospital mortality, odds ratio (OR) 0.70 (95%CI 0.65, 0.76). Major predictors of 30-day in-hospital mortality were acute illness severity score (AISS) OR 6.9 (95%CI 5.5, 8.6) and comorbidity score OR 2.4 (95%CI 1.2, 4.6). At a median follow-up of 5.9 years post-discharge, 19.0% had died. The strongest long-term predictor of mortality was admission AISS OR 6.7 (95%CI 4.6, 9.9). The overall survival half-life after hospital discharge was 16.6 years. Survival was significantly worse for weekend admissions at 20.8 years compared to weekday admissions at 13.3 years.

**Conclusion:**

Weekend admission of acute medical patients is associated with reduced 30-day in-hospital mortality but reduced long-term survival.

## Introduction

Over the past two decades, there has been a growing interest in weekend medical emergency admissions and mortality, with the conclusion that any increase in mortality might be attributable to a reduction in staff cover (including physician coverage) [1–3] at weekends, with a shift towards more junior, less experienced medical staff [4], and also reduced access to certain treatments and procedures [5–9]. Mortality has been estimated on average to be 10% higher at the weekend [1, 5, 7–15] perhaps related to staff or organisational rather than patient factors. Although it may appear reasonable to implicate the reduced availability of diagnostic procedures and therapeutic modalities at weekends, studies have failed to link higher weekend mortality rates to delays in procedure availability and timeliness [16, 17]. It has also been suggested that the case-mix could be different for weekend emergencies [1, 8, 9].

In this study, we assess all acute medical admissions to St James’s Hospital between 2002 and 2023. We investigate 30-day in-hospital and long-term mortality and the predictors of these, including the influence of weekend admission.

## Methods

### Background

St James’s Hospital, Dublin serves as a secondary care centre for emergency admissions in a catchment area with a population of 270,000 adults. All emergency medical admissions are admitted from ED to an Acute Medical Admission Unit (AMAU), the operation and outcome of which have been described elsewhere [18, 19].

### Data collection

An anonymous patient database was employed, assembling core information from each clinical admission including details from the patient administration system, national hospital in-patient enquiry (HIPE) scheme, the patient electronic record, and laboratory data. HIPE is a national database of coded discharge summaries from acute public hospitals in Ireland. For diagnosis and procedure coding, the International Classification of Diseases (9th Revision ICD-9-CM) was employed < 2005 with ICD-10-CM thereafter. Data included parameters such as the unique hospital number, admitting consultant, date of birth, gender, area of residence, principal and up to nine additional secondary diagnoses, principal and up to nine additional secondary procedures, and admission and discharge dates. Additional information cross-linked and automatically uploaded to the database includes physiological, haematological, and biochemical parameters. This study includes all acute medical admissions admitted in the 21 years between 2002 and June 2023, with follow-up on the Irish National Death Register to December 2021. The analysis of weekend vs weekday outcomes utilised two sets of consecutive days—Saturday/Sunday were compared with Tuesday/Wednesday. The representative weekdays chosen were selected as those being at least risk of a weekend effect influence. This study received institutional Ethics Committee approval.

### Risk predictors

Derangement of admission biochemical parameters may be utilised to predict clinical outcome. We have previously derived and applied an AISS [20], predicting 30-day in-hospital mortality from parameters recorded in the ED. A weighted age adjusted score was derived; six risk groups (I–VI) were identified with initial cut-points for 30-day in-hospital mortality set at 1, 2, 4, 8, and 16%.

Comorbidity was assessed by the previously described comorbidity score [21]. To devise the score, we searched ICD codes that captured chronic physical or mental health disorders that limit people in activities that they generally would be expected to be able to perform were grouped according to the following ten systems: (i) cardiovascular, (ii) respiratory, (iii) neurological, (iv) gastrointestinal, (v) diabetes, (vi) renal, (vii) neoplastic disease, (viii) others (including rheumatological disabilities), (ix) ventilatory assistance required, and (x) transfusion requirement. In addition, we searched our hospital’s other databases for evidence of diabetes (Diamond database) [22], respiratory insufficiency (FEV1 < 2 L), troponin status (high sensitivity troponin ≥ 25 ng/L) [23], low albumin (< 35 G/dL), and anaemia (haemoglobin levels < 10 G/dL) or chronic renal insufficiency, MDRD < 60 mL/min*1.73 m^2^. Each component of the score was then weighted according to 30-day in-hospital mortality.

### Match to Irish National Death Register

Data analysis uses anonymised data, via an instruction set (Stata code) to assemble and analyse data in computer memory from various discrete files, patients in each being identified by a unique hospital admission number or identifier and the series number (case ID, related to the admission date/time). Each patient address was accessed once to derive the Deprivation index [24]. The match to the Irish National Death Register and hospital patient administration record involved cleaning of string data and merge of the databases (matched on date of birth and surname). Gender mismatches were deleted, and string-based fuzzy matching techniques calculated a score of text agreement (both name/surname and address) similarity. The match was confirmed by visual inspection. A small number of residual observations (~ 350) were manually accepted or rejected, using online death notices. The information then, on the database, consist of 0/1 and date of death to preserve the anonymous database nature.

### Statistical methods

Descriptive statistics were calculated for background demographic data, including means/standard deviations (SD), medians/inter-quartile ranges (IQR), or percentages. Comparisons between categorical variables and mortality were made using chi-square tests. We adjusted the outcome computation for other known predictor variables including AISS [20, 25], comorbidity score [21], and blood culture status [26]. We employed a logistic model with robust estimates to allow for clustering; the correlation matrix thereby reflected the average discrete risk attributable to each of these predictor variables [20].

Logistic regression analysis identified potential mortality predictors and then tested those that proved to be significant univariate predictors (*p* < 0.1 by the Wald test) to ensure that the model included all variables with predictive power. We used the margins command in Stata to estimate and interpret adjusted predictions for sub-groups, while controlling for other variables such as time, using computations of average marginal effects. Margins are statistics calculated from predictions of a previously fitted model at fixed values of some covariates and averaging or otherwise over the remaining covariates. In the multivariable logistic regression model, we adjusted univariate estimates of effect, using the previously described outcome predictor variables. Survival regression calculations were undertaken employing the non-parametric Kaplan–Meier model. This non-parametric method does not assume the distribution of the outcome variable, with the Kaplan–Meier curve illustrating the change in survival probabilities over time.

Adjusted odds ratios (OR) and 95% confidence intervals (CI) were calculated for those predictors that significantly entered the model (*p* < 0.10). Statistical significance at *p* < 0.05 was assumed throughout. Stata v.17.0 (Stata Corporation, College Station, Texas) statistical software was used for analysis.

## Results

### Patient demographics

The total cohort of acute medical admissions, between 2002 and June 2023, consisted of 187,553 admissions in 90,848 patients. This included patients admitted directly into the Intensive Care Unit or High Dependency Unit. The proportion of males was 51.6%. The median (IQR) length of stay (LOS) was 5.0 (1.8 11.1) days. The median (IQR) age was 64.1 (46.3, 77.7) years, with the 90th percentile boundary at 85.4 years.

For those followed up post-discharge, with a median risk exposure time of 33 months, there were 12,492 deaths in 88,869 subjects; this 14% death rate calculated to survival of 12.6 years. A longer follow-up from the first decade, with median risk exposure time of 62 months, revealed 9790 deaths in 50,674 subjects. This rate of 19.3% calculated a survival half-life of 16.7 years. Compared with the population survival of 22.2 years at median age of 62, this emergency admission group has a shortfall of range 5.5–9.6 years of life expectancy.

In the combined Tuesday/Wednesday and Saturday/Sunday cohort, there were 109,232 admissions in 57,059 patients (Table 1). The demographics showed proportion of males 51.8%. The median (IQR) LOS was 5.0 (1.9, 11.5) days. The median (IQR) age was 64.0 (46.1, 77.7) years, with the 90th percentile boundary at 85.4 years. The demographics for the Tuesday/Wednesday and Saturday/Sunday subgroups were similar to the combined cohort (Table 1).

**Table 1:** Acute medical admissions (2002–2023): admission characteristics

Variable		Tuesday/Wednesday	Saturday/Sunday	*p*-value
Admissions		55,249	53,983	
Age (years)		64.5 (46.3, 78.0)	63.6 (45.8, 77.4)	< 0.001
LOS (days)		5.3 (2.0, 12.0)	5.0 (1.5, 11.0)	< 0.001
Gender	Male	27,975 (50.6%)	27,972 (51.8%)	< 0.001
	Female	27,274 (49.4%)	26,011 (48.2%)	
Acute illness	1–3	16,013 (29.0%)	16,549 (30.7%)	< 0.001
Severity score	4	9151 (16.6%)	9236 (17.1%)	
	5	10,142 (18.4%)	10,084 (18.7%)	
	6	19,943 (36.1%)	18,114 (33.6%)	
Charlson Index	0	26,413 (47.8%)	28,064 (52.0%)	< 0.001
	1	13,625 (24.7%)	15,048 (27.9%)	
	2	15,211 (27.5%)	10,871 (20.1%)	
Comorbidity score	< 6	37,366 (67.6%)	35,732 (66.2%)	0.004
	6 < 10	15,575 (28.2%)	16,098 (29.8%)	
	≥ 10	2308 (4.2%)	2153 (4.0%)	

### 30-Day in-hospital mortality

The overall 30-day in hospital mortality rate in the 21-year study period was 3.2% (95%CI 3.1%, 43.3%). Unadjusted 30-day in-hospital mortality for weekday admissions was 3.8% (95%CI 3.7–4.0%) vs 2.6% (95%CI 2.5–2.7%) for weekend admission. This revealed a favourable impact (‘*t*’ Scheffé – *F* = 121.8: *p* = 0.000) of weekend admission on 30-day in-hospital mortality.

The multivariable logistic regression model of 30-day in-hospital mortality, adjusted for major outcome predictor variables (Table 2), not alone demonstrated no evidence of an adverse weekend effect but a substantially lower mortality OR 0.70 (95%CI 0.65, 0.76). Major outcome predictors were the AISS, OR 6.89 (95% CI 5.51, 8.60) and the comorbidity score, OR 2.35 (95%CI 1.20, 4.58). Age > 70 years, Charlson Index, and MDC categories of Respiratory and Neurology were also significant predictors of 30-day in-hospital mortality (Table 2).

**Table 2:** Multivariable logistic predictors of 30-day in-hospital mortality

Variable	OR	Std. Err	*z*	*p* >|*z*|	[95% Conf. interval]	
Weekend	0.70	0.03	− 9.3	0.00	0.65	0.76
AISS	6.89	0.78	17.0	0.00	5.51	8.60
Comorbidity score	2.35	0.80	2.5	0.01	1.20	4.58
Charlson Index	1.56	0.04	17.9	0.00	1.49	1.64
Respiratory	1.43	0.07	7.7	0.00	1.31	1.57
Cardiovascular	1.08	0.06	1.4	0.15	0.97	1.21
Neurology	1.79	0.11	9.7	0.00	1.59	2.01
Age > 70 years	1.70	0.07	12.6	0.00	1.56	1.85

### Long-term mortality

For the comparator cohorts, the total of deaths, in hospital within 30-days of admission, was 6145 (18.5% including readmissions). There were 13,781 additional deaths in follow-up period. The survival half-life post-discharge of both cohorts, weekday and weekend, approximated to 16.6 years.

Patients admitted over a weekend had significantly worse long-term survival, 1.76 (95%CI 1.66, 1.85). After adjustment for other major predictor variables, the effect was more marked: OR 1.98 (95%CI 1.88, 2.10) (Table 3). The strongest long-term predictive variables were the admission AISS, OR 7.70 (95%CI 6.29, 9.43) and Charlson Index OR 1.33 (95%CI 1.28, 1.38) and Older (age > 70) OR 1.98 (95%CI 1.88, 2.10). Comorbidity score 1.16 (95%CI 1.15, 1.17) remained predictive, albeit attenuated.

**Table 3:** Cox proportionate hazard regression model of long-term mortality

Variable	Hazard ratio	Std. Err	*z*	*p* >|*z*|	[95% Conf. interval]	
Weekend admission	1.98	0.06	24.2	0.00	1.88	2.10
AISS	7.70	0.80	19.7	0.00	6.29	9.43
Comorbidity score	1.16	0.01	30.3	0.00	1.15	1.17
Charlson Index	1.33	0.02	15.9	0.00	1.28	1.38
Respiratory	1.12	0.04	3.4	0.00	1.05	1.20
Cardiovascular	1.22	0.05	5.1	0.00	1.13	1.31
Neurology	1.08	0.04	2.1	0.04	1.00	1.16
Age > 70 years	1.98	0.06	24.2	0.00	1.88	2.10

**Fig. 1:**
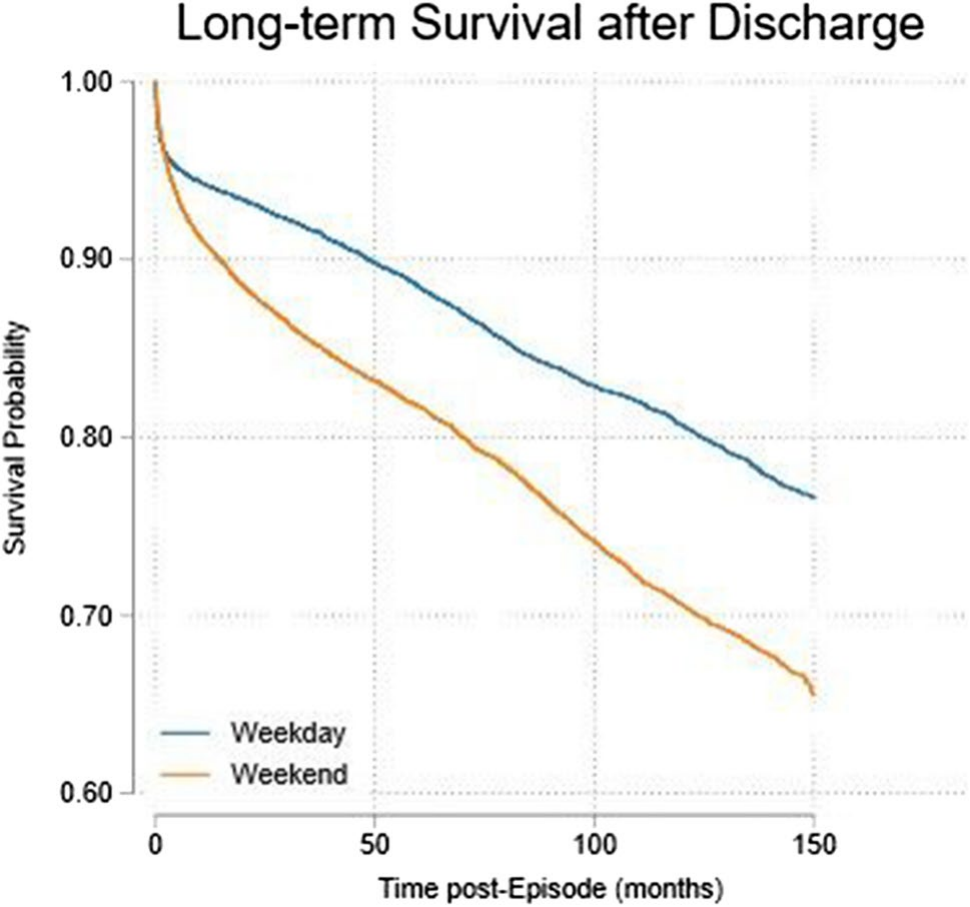
Follow-up to 71 months, analysed with the Kaplan–Meier survival estimate

We assessed long-term survival, employing the a non-parametric product-limit Kaplan–Meier approach, that makes no assumption regarding the distribution of the outcome variable (i.e., time). We undertook survival analysis for admissions between 2002 and 2018 inclusive; this yielded a median follow-up observation period of 5.9 years. Of 29,917 patients admitted over that period, 19.0% had died. The survival half-live was projected at 16.6 years. The Irish Central Statistics Office reference survival for this population, of average age 60, is 20.5 years. The weekday survival half-life of 249 months (20.8 years) was better than for the weekend admissions of 159 months (13.3 years) (Fig. 1).

## Discussion

These data provide insight into the acute and longer-term survival of patients following an acute medical admission. Our data revealed that weekend admission was associated with 30% lower likelihood of 30-day in-hospital mortality but a 7.5-year reduction in long-term survival. Short-term mortality was best predicted by admission illness severity and comorbidity, in line with our previous findings. Long-term mortality was best predicted by illness severity at the index admission.

These data confirmed our earlier observations that illness severity as reflected by the AISS at time of admission might if altered at the weekend provide some explanation as to how the putative 10% excess mortality for weekend admissions cited by other sources might operate [1, 5, 7–11, 13–15]. The poor long-term survival of the weekend cohort was remarkable for its effect size, contrasting with that of the weekday admitted cohort. This represented a shortfall of 36.1% in life expectancy. There appears no more likely hypothesis than that the weekend cohort represented an intrinsically higher risk group, although this was not identified by the baseline factors measured in this study.

There are a number of possible explanations for this that could be explored in further studies. Individuals with greater levels of deprivation or with greater social isolation may be more likely to present at weekends; this group may have worse long-term outcomes due to these factors. Patients admitted over the weekend may be less likely to receive optimisation of secondary prevention, appropriate clinical follow-up arrangements, or integration of care with their primary care provider. While not immediately intuitive how this could be related, there are two potential explanations; a small number of patients may be discharged quickly over the same weekend, but the more probable association may involve the fact that due to work patterns, the majority of the weekend care team will not be those involved in the further hospital management and discharge of the patient. Future studies could explore these factors in greater detail.

The weekday differs from the weekend in that ordinarily all staff are present during the week. This provides a resource base with a high presence of expertise and options to manage care expeditiously weighted by expert input. Any departure from this optimum, with either a reduction of the total resource available or an increase patient risk profile (occurring for whatever reason), can be expected to trend towards worse outcomes. Maggs et al. [5] examined outcomes for each day of the week, and also at nights and for out-of-hours periods; total mortality was increased for admissions on Mondays, at night, and in all out-of-hours periods. These authors concluded a likely explanation involved both illness severity and immediately available local resources. This finding was consistent with our prior conclusions that the increased mortality associated with a weekend emergency admission to be due to increased illness severity and altered case-mix [27]. Any formal analysis needs to consider intrinsic difference in baseline risk characteristics before attribution of different outcomes to patient specific factors; Froom and Shimoni [28] have demonstrated that electronically retrieved laboratory data might be used as a potential quality indicator to predict mortality in internal medicine departments. The ‘July effect’ examined the first day of new staff, the first Wednesday in August have a higher early death rate in English hospitals compared with the prior more experienced-of-year staff on the previous Wednesday [29]. Emergency PCI procedures undertaken at night were associated with a significant time-dependent effect with increased risk of an in-hospital deaths [30]; there is an additional factor of the well-known circadian variation for deaths from ischaemic heart disease. Of course, this is not to dismiss the importance of potential compromise of care because the level of staffing in the hospital is often lower than during the week. Data from internal medicine in Spain for 429,880 adults with a presentation and emergency medical admission confirmed a higher admission on weekends is associated with higher mortality than admission during the week [31]. Our data provides more insight, with the focus on remote late date mortality in the community for weekend admissions that are very unlikely to have much if anything to do with the hospital care.

As with any study design, there are certain limitations inherent in our current work. The advantages are the long-term nature of the observations; the increased sample size has reduced the errors around estimates of prognostic variables. In terms of the short-term weekend mortality, our earlier data was neutral not showing a weekend effect with worse outcomes [27, 32], unlike the experience of others [1, 5, 7, 8, 10, 11, 13–15]. The increased sample size has now demonstrated a lower short-term mortality for weekend admissions. Our AMU development imposed system controls on the emergency admission process that dramatically improved outcome; outcomes generally globally have improved with strategies suggested to improve the quality of care [10, 11, 19]. Therefore, the current position with improved structures [33] and the presence of senior consultant [34] surveillance may be expected to improve outcomes. However, our finding that matters have progressed to the position with a lower hospital mortality following a weekend admission is quite unexpected.

There are caveats to the current analysis. To ensure adequate risk exposure, the first 10 years of admissions between 2002 and 2011 are overweighted in the analysis; the 5.9-year median exposure means that the majority of the second decade of admissions do not contribute to the survival analysis. The calculated overall survival half-life overall is a best estimate and dependent on the point at which the estimate is made on the survival curve. However, the data, notwithstanding this limitation, reveals very different survival between those admitted during the weekend compared with the weekday.

In conclusion, we have demonstrated a lower 30-day in-hospital mortality for weekend admissions but paradoxically a significant reduction in long-term survival for the same cohort.

## Data Availability

No further data is available for sharing. All pertinent data has been published in this manuscript.
